# WE FOUND DIGITAL, WHERE IS INCLUSION?: TOPIC MODELING OF DIGITAL INCLUSION STUDIES

**DOI:** 10.1093/geroni/igae098.0834

**Published:** 2024-12-31

**Authors:** Ha Neul Kim, Manjun Kim

**Affiliations:** Michigan State University, East Lansing, Michigan, United States; Florida State University, Gyeonggi-do, Kyonggi-do, Republic of Korea

## Abstract

An increasing number of scholars agree that digital inclusion is to mitigate the digital divide and enhance social inclusion. However, since there is no consensus on the definition of digital inclusion, as of now, it is important to explore key concepts of digital inclusion studies to better understand what constitutes digital inclusion. The study employed a topic modeling methodology to analyze extensive collections of abstract data. Topic modeling methodology was applied to analyze vast amounts of abstract data. In total, 1,741 abstracts from book chapters, journal articles, and conference papers published by 2023 were analyzed. Data pre-processing included deleting stopwords, determining synonyms and compound words, extracting nouns, and identifying keywords based on word frequency and co-occurrences. A co-occurrence matrix was used to generate semantic networks, and community detection was applied based on modularity, the measurement of closeness within and distance across keyword groups. This study showed that there are six themes under digital inclusion studies: Policy and systematic intervention for digital inclusion (29.5% of keywords), Technology experience for older adults (27.87%), Digital divide in accessibility (16.05%), Online education and training resources (10.85%), Delivery of healthcare and equity (10.85%), and Digital inclusion in business and market (4.88%). The biggest cluster showed how digital inclusion is not only a matter of having access online or learning digital literacy but a systematic concern that involves policy intervention. However, unlike the name referring to certain ‘inclusion’, the topics that were studied so far, yet built social domain.

